# Hyperglycemia Identification Using ECG in Deep Learning Era

**DOI:** 10.3390/s21186263

**Published:** 2021-09-18

**Authors:** Renato Cordeiro, Nima Karimian, Younghee Park

**Affiliations:** Department of Computer Engineering, San Jose State University, San Jose, CA 95119, USA; renato@renatocordeiro.com (R.C.); younghee.park@sjsu.edu (Y.P.)

**Keywords:** electrocardiogram, artificial neural networks, deep learning, glucose, hyperglycemia, machine learning

## Abstract

A growing number of smart wearable biosensors are operating in the medical IoT environment and those that capture physiological signals have received special attention. Electrocardiogram (ECG) is one of the physiological signals used in the cardiovascular and medical fields that has encouraged researchers to discover new non-invasive methods to diagnose hyperglycemia as a personal variable. Over the years, researchers have proposed different techniques to detect hyperglycemia using ECG. In this paper, we propose a novel deep learning architecture that can identify hyperglycemia using heartbeats from ECG signals. In addition, we introduce a new fiducial feature extraction technique that improves the performance of the deep learning classifier. We evaluate the proposed method with ECG data from 1119 different subjects to assess the efficiency of hyperglycemia detection of the proposed work. The result indicates that the proposed algorithm is effective in detecting hyperglycemia with a 94.53% area under the curve (AUC), 87.57% sensitivity, and 85.04% specificity. That performance represents an relative improvement of 53% versus the best model found in the literature. The high sensitivity and specificity achieved by the 10-layer deep neural network proposed in this work provide an excellent indication that ECG possesses intrinsic information that can indicate the level of blood glucose concentration.

## 1. Introduction

With the advent of smart computing sensors (smart smartwatches, smartphone, and wearable, etc.) and the emergence of the internet of medical things (IoMT), innovative healthcare solutions and services have become more important than ever. One of the application of IoMT is to enable smart wearable devices for continuous and remote monitoring of people’s health to improve healthcare management in a variety of hospital and home environments. Moreover, IoMT devices such as Apple Watch and Fitbit not only became ubiquitous in a user’s daily life but also are becoming smarter through embedded sensors that can monitor different physiological signals. For instance, Apple Watch series 4 and 5 have added electrodes to the digital crown and on the back of the watch that can collect cardiac activity (ECG signal) to detect heart arrhythmia.

ECG has been primarily used in cardiac and medical fields but its commercial potential is now being explored in a variety of interesting ways. For example, as previously mentioned, smart watches are now reading ECG signals to diagnose cardiac diseases, which are the leading causes of death in the world. According to the center for disease control and prevention (CDC), approximately 655,000 people die of heart disease in the U.S. every year [1]. Over the last few decades, there has been an increasing effort to develop computer-based automatic diagnostics of the ECG [2,3,4,5]. More recently, ECG has been used for hyperglycemia detection and continuous glucose monitoring [6,7,8]. Hyperglycemia represents high level of glucose in the blood and prolonged or abnormal events of hyperglycemia is common in people with diabetes. The number of people with diabetes has increased from 108 million in 1980 to 422 million in 2014, according to World Health Organization (WHO) [9]. In the U.S. alone 30 million adults are with diabetes, from which 7.2 million are not aware that they suffer from that condition [10]. Diabetes and prolonged hyperglycemia, when not treated, can lead to serious health problems including blindness, limb amputation, heart diseases, and even death. Currently, the COVID-19 pandemic has highlighted the impact of comorbidities such as diabetes to the virus fatality rate [11]. Going further, Ref. [12] showed that high level of blood glucose at time of admission is an independent predictor of mortality even in COVID-19 patients without diabetes. In order to reduce the serious consequences caused by diabetes and hyperglycemia, continuous monitoring of blood glucose concentration should be used, especially by those who manage blood glucose levels with insulin, requiring manual injections or the use of insulin pumps.

**Figure 1:**
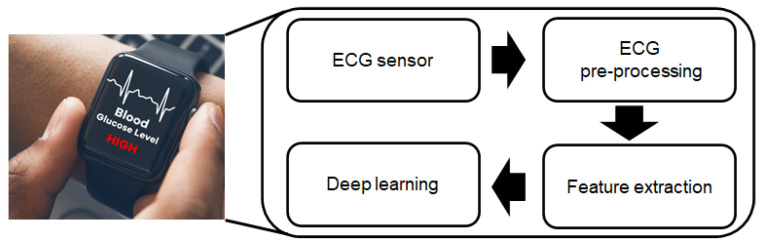
Illustrative schematic of proposed ECG-based hyperglycemia detection.

In this paper, we go beyond the use of ECG for heart disease and aim for a non-invasive method for continuous glucose concentration. This paper provides a detailed explanation of the effects hyperglycemia has on ECG signals and explores a new method to allow us to measure and detect them through a non-invasive technique. People can take advantage of technology that they are already wearing in order to track their glucose level concentration. Our high level proposed deep learning architecture with the state-of-the-art feature extraction techniques is illustrated in Figure 1, which can be adopted into different ECG domain applications.

Our main contributions are described as follows:

- We develop a 10-layer deep learning based hyperglycemia detection technique and more robust approaches for processing ECGs.
- We present different feature extraction techniques. Specifically, we investigate novel fiducial methods such as slope and temporal and amplitude characteristics. This resulted in a feature size reduction of 97% when compared to a full ECG cardiac cycle.
- To demonstrate the effectiveness, robustness, and generalization ability of our proposed methods, we conducted experiments on a new ECG database containing 68,274 samples collected from 1119 subjects.
- We provide detailed classification analysis of age, weight, height, and heart rate and discuss the impact of these on hyperglycemia.

The remainder of the paper is organized as follows. Section 2 describes the background of hyperglycemia and ECG. Literature review presented in Section 3. In Section 4, we discuss the proposed approach including pre-processing steps and feature extraction technique. Section 5 details the models, simulations, and metrics used. The experimental results are shown in Section 6. Finally, we conclude the paper in Section 8.

## 2. Background

### 2.1. Hyperglycemia

Hyperglycemia means high (hyper) glucose (gly) in the blood (emia), also commonly referred to as high blood sugar, and is a state of elevated levels of glucose in the bloodstream. This naturally occurs following meals, but after a few hours the body should go back to a normoglycemia state due to work of the insulin hormone [13]. More importantly, high blood glucose concentration and diabetes mellitus are important predictors of sudden cardiac arrest, which itself has close relationship with ECG [14]. The limit between normoglycemia and hyperglycemia can vary from health organizations, but it is usually 100 mg/dL under a fasting state. A person in a constant state of hyperglycemia, especially when fasting, can be in a situation where the body is not able to handle glucose anymore. This is usually a consequence of insulin resistance, a condition where the body becomes insensitive to insulin and, thus, the hormone is not able to decrease the glucose concentration as it used to do. Insulin resistance is considered one of the pathways to type-2 diabetes mellitus [15].

The traditional method for hyperglycemia evaluation is assessment of glucose concentration in blood samples. Samples can be a few drops acquired by pricking the finger or larger amounts usually obtained by health professionals. Both processes are invasive, which is associated with pain and discomfort, being a barrier for their widespread use as a screening mechanism. Another often under-recognized but still critical problem is the possibility of bloodborne pathogen transmission [16] such as hepatitis B/C virus (HBV/HCV), human immunodeficiency virus (HIV), and others, due to the sharing of the same device or its accessories among infected people. The paper from [17] states that 15 out of 18 HBV infections outbreaks since the year 1990 were associated to the improper use of blood glucose monitoring systems. Lastly, the use of invasive glucose monitoring systems also creates a significant environmental impact due to the creation of medical waste [18]; therefore, a non-invasive mechanism to detect hyperglycemia would be useful for individuals and the society in general.

### 2.2. Electrocardiogram (ECG)

ECG signals have several essential components that are recorded as a series of positive and negative waves referred to as the P wave, QRS complex, and T wave. The P peak of the normal heartbeat is a small upward wave, which indicates atrial depolarization. Approximately 160 ms after the onset of the P wave, the QRS wave is caused by ventricle depolarization. Finally, one observes the ventricular T wave in the electrocardiogram, which represents the stage of repolarization of the ventricles [19].

## 3. Literature Review

Non-invasive technologies for measuring glucose levels have been researched for decades, being the first promising research performed by Kost et al. [20] in the year 2000. Since then new technologies and approaches have been tried and, although none are consumer-ready, great advances have been made. Afterward, a lot of effort has been devoted to develop a non-invasive sensor using different technologies such as photoacoustic spectroscopy, Raman spectroscopy, electrochemical reaction, ultrasound, and many others; most of them based on spectroscopy, which explores the intrinsic relation between electromagnetic radiation and the underlying material [18]. Although very promising, the use of spectroscopy for glucose estimation has still not reached a cost-effective level, sometimes requiring expensive custom hardware. Reddy et al. [21] designed a device to monitor blood glucose level using near infrared sensors. Julian et al. [22] also proposed a near infrared sensor to measure glucose concentration. Pai et al. [23] implemented FPGA-based glucose monitoring with photoacoustic signal amplitude. Anas et al. [24] measured the blood electrical impedance in which the electrode was made using four tin-lead solder electrodes. They evaluate their hardware by measuring data over 10 subjects, ages between 20–25 years old. Liu et al. [25] developed a 4E-AFE and AD5933 measurement circuit to measure contact impedance between electrodes and the skin for glucose monitoring. Vilaboy et al. [26] used Raman spectroscopy to measure glucose. Even though some work proposed non-invasive glucose monitoring, they are not developed for continuous monitoring in which users can see their glucose level anytime at a glance.

Other works have shown that blood heart rate variability (HRV) can be modulated by blood glucose levels [27]; therefore, several studies investigating HRV and glucose levels have been developed. Amanipour et al. [27] analyzed the HRV frequency domain components of a 59-year old diabetic female subject under normoglycemic and hyperglycemic conditions. They noticed a 6-fold decrease in the low frequency/high frequency ratio. Although the study was limited to just 1 person, it corroborated the results achieved by Fujimoto et al. [28] where that ratio was first identified to be negatively correlated with blood glucose concentration. In 2017, Perpiñan et al. [29] assessed the impact of taking a 75 g oral glucose testing in 25 subjects, 15 with metabolic syndrome, and 10 as a control group. They identified that the metabolic syndrome subjects presented significant higher HRV irregularity than the control group after 30 min of glucose intake. After 60 min of glucose intake, the HRV irregularity in people with metabolic syndrome decreased, but that was not observed in the control group.

Another metric known to be impacted by blood glucose level is the *QT* interval, which is assumed to be prolonged on people with metabolic syndrome due to their difficulty in metabolizing glucose [30]. Farina et al. [31] assessed 99 subjects, 34 healthy, 32 pre-diabetic, and 33 type-2 diabetics and noticed a prolongation in the *QT* interval of pre-diabetic subjects. Due to the small number of subjects, this result was not considered statistically significant and the authors suggested an analysis on a large population. Suys et al. [32] monitored the ECG and blood glucose concentration of 9 type-1 diabetic children by using a Holter and continuous glucose monitoring device. They also identified a prolongation of *QT* and *QTc* (*QT* interval adjusted by Bazett formula) with lower blood glucose concentration. Marfela et al. [33] also studied the impact of blood glucose in *QT* duration. More specifically, they analyzed 20 healthy subject (10 men and 10 women) and noticed that acute hyperglycemia resulted in a significant increase not only in *QTc* interval and *QTc* dispersion but also in *PR* interval. The prolongation of the PR interval was something never investigated before as related to glucose concentration. Different from almost all other studies, Marfela et al. [33] analyzed healthy subjects instead of subjects with diabetes, metabolism syndrome, or other health issues. Nguyen et al. [34] analyzed the effect of hypoglycemia and hyperglycemia on ECG parameters, including *HR*, $QT_{c}$, *PR*, $RT_{c}$, and $T_{p}T_{ec}$ (T-peak to T end). They identified that low blood glucose was associated with prolonged $RT_{c}$, $QT_{c}$, and $T_{p}T_{ec}$, but not with *PR*, in contradiction to Marfela et al. [33] findings. In contrast, high blood concentration was related not only to a decreased $RT_{c}$, $QT_{c}$, and $T_{p}T_{ec}$, but also to an increased *PR*. Their study was limited to five subjects and, as noted by the authors, could benefit from larger population study. All the aforementioned research focused mainly on identifying the impact of glucose concentration in different features captured by an ECG. In 2014, Nguyen et al. [6] went one step further by proposing a neural network model to detect hyperglycemia using an ECG. They proposed a 3-layer feed ANN with one input layer, one hidden layer, and one output layer, with Tansig as the transfer function between the hidden and output layer. In addition, 16 features extracted from 10 adolescent’s ECG signal are summarized in Table 1. The time and frequency domain features used were selected from the Task Force work that created guidelines for HRV analysis [35]. The dataset was divided in to 35% for training, 35% for validation, and 30% for testing. The ANN model was tested using five different optimizers and the resulting sensitivity and specificity were compared. The best model was a 3-layer ANN using Levenberg–Marquard algorithm with nine nodes in the hidden layer, which provided a sensitivity of 70.59% and specificity of 65.38% in the testing dataset; however, the prior approach is limited in terms of data size and performance. To overcome some of the limitations of that study, we introduce a new deep learning architecture along with feature extraction techniques to improve performance. Our proposed work was tested with private database that contains ECG signals of 1119 subjects.

## 4. The Proposed Approach

**Figure 2:**
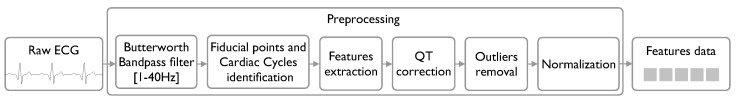
Preprocessing steps.

In this section, we describe our proposed process of an ECG-based hyperglycemia detection. Figure 2 illustrates the steps that an ECG goes through in order to provide the features used in the deep learning model. Those steps include *Filtering, Segmentation, Feature extraction, *QT* correction, Outlier removal, and Normalization* and are detailed below.

### 4.1. Filtering

ECG contains various types of noise sources, including baseline wander (BW), motion artifact (MA), and electrode movement (EM). These noises can degrade the accuracy of the fiducial features detection algorithm. In order to remove eventual artifacts due to the setup and removal of the electrodes on the subject, the first and last 10 s of the raw ECG signals are ignored. The remaining data are filtered using Butterworth bandpass filter order 4 and a frequency range of 1 Hz to 40 Hz [36]. BioSPPY library [37] was used to perform the filtering.

### 4.2. Fiducial Points and Cardiac Cycles Identification

The next step after filtering is to process the signal to identify the cardiac cycles. This is important due to our choice of use fiducial features from cardiac cycles as our deep learning model input (as further detailed in the next step). The process starts by identifying the R-peaks, which allow us to to segment individual heart beats and further analyze the cycle for the remaining waves. R-peak detection can be performed using the Pan–Tompkins [38] algorithm and we used a modified version of that algorithm, which is implemented in the BioSPPY library. The remaining waves— P,Q,S,T—were then identified with the help of the NeuroKit library [39].

### 4.3. Features Extraction

There are several different methods for features extraction from ECG signals, ranging from simple and direct measurements based on fiducial points to more complex ones that are based on the entire wave morphology [40]. The former case includes amplitudes and distances between points and is known as a fiducial-based method. The latter includes wave frequencies such as discrete wavelet transform (DWT) coefficients and is categorized as a non-fiducial-based method. In this work, several experiments were performed by testing a variety of fiducial-based features that could provide similar or better performance than using the whole cardiac cycle data (one heartbeat) as the model input. A collection of 18 features composed of 9 fiducial distances directly connecting different fiducial points and the respective slopes of such lines were found to accomplish that objective. The decision to classify each heartbeat independently provides us with a significance increase in the size of the training and testing dataset. Another advantage is that it decreases the potential for overfit since even sequential heartbeats from a same subject presents different fiducial points values while the classification in hyperglycemia/non-hyperglycemia is still the same. Features that depends on more than a single heartbeat, such as HRV, were not used since all features in this work must be obtained from within a heartbeat.

**Figure 3:**
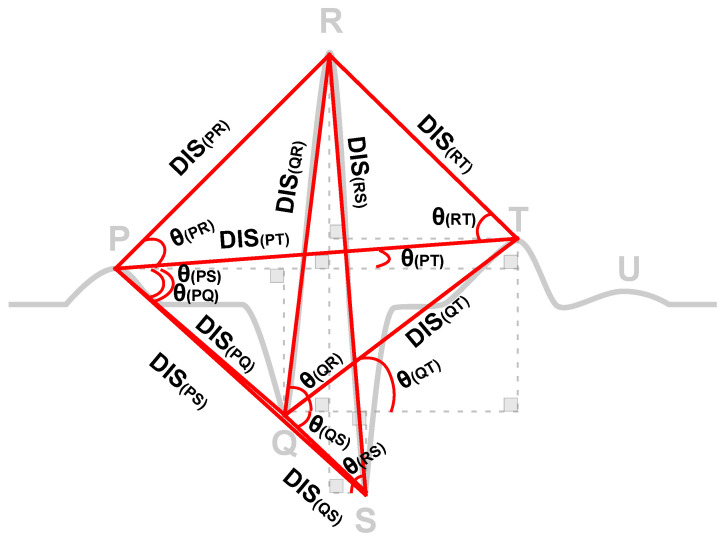
Features extracted from an ECG cycle.

Initially, the heartbeat (one cycle of ECG) is quite large (600 data points). This fact may make subsequent computations difficult. Moreover, it may include redundant and useless information due to correlation and inter-dependencies between data points where the informational trait of each heartbeat and its contribution to the distinction of classes vary significantly. The aforementioned 18 novel fiducial features are potentially a distinct ECG characteristic leading to the best results. An illustration of the distances and slopes used can be seen in Figure 3.

The direct line length between two points, such as *P* and *Q*, were calculated using Euclidean distance as shown in Equation (1).

(1) $$\begin{array}{c} \begin{array}{cc} distance(P,Q) & =\sqrt{PQx^{2}+PQy^{2}}\\ \; & =\sqrt{{\left(Qx-Px\right)}^{2}+{\left(Qy-Py\right)}^{2}} \end{array} \end{array}$$

where the Qx and Px show the location of fiducial *Q* and *P* at *x*; Qy and Py are indicate the amplitude of of fiducial *Q* and *P*.

The slope was calculated using Equation (2):

(2) $$slope\left(PQ\right)=\frac{PQy}{PQx}=\frac{Qy-Py}{Qx-Px}$$

The list of all 18 features used in this work is shown in Table 2.

### 4.4. QT Correction

The *QT* interval is a metric that is known to have its value impacted by the subject’s heart rate [41] and from glucose concentration [33]; therefore, to help the model better detect the glucose impact, it is important to have the heart rate interference reduced as much as possible. Several formulas have been developed to correct the *QT* interval for heart rate, the famous being Bazett’s equation. While still widely used, it does not always work efficiently since it over-corrects when heart rate is lower than 60 bpm and under-corrects when heart rate is higher than 60 bpm. A study performed by Vandenberk et al. [42] analyzed five different *QT* corrections equations, including Bazett’s. Their conclusion was that the Framingham formula provided the best correction, so that was used in this work. Based on that formula, the *QT* interval can be converted by using Equation (3):

(3) QTc=QT+0.154∗(1−RR)

where QTc is aligned *QT* interval and *RR* is calculated from the heart rate as shown in Equation (4):

(4) $$RR=\frac{60}{heartrate}$$

### 4.5. Outliers Removal

The features extracted in the previous step, can present some outliers. Removal of such inconsistent data contributes to a faster training and better model performance. The outliers were identified using the interquartile range (*IQR*) [43] method, which defines a lower and upper bound based on the range between the first and third data quartiles. Equation (5) below shows how *IQR* and the lower and upper bounds are calculated:

(5) $$\begin{array}{c} \begin{array}{cc} IQR & =Q3-Q1\\ Lowerbound & =Q1-1.5*IQR\\ Upperbound & =Q3+1.5*IQR \end{array} \end{array}$$

where *Q*1 and *Q*3 are the first quartile and third quartile values, respectively. Data points located below the lower bound or above the upper bound values were removed. A high number of outliers could be a consequence of a very noisy signal or also a poor fiducial points identification algorithm; therefore, it is important to not only remove that data but also check the percentage of the whole data that were flagged as outliers. High percentages may indicate a need for a revision of the fiducial points identification algorithm. The dataset used in this work had 68,274 samples and 16,756 were identified as having at least one outlier feature and thus were removed, leaving 51,518 to be used in the model. Removing the entire sample due to just one outlier feature is a very conservative approach and was used since the number of samples left was still significant and more than enough for training and testing. If a smaller dataset was used, different outlier removal approach could be used such as replacing the outlier feature with the average value of that feature for that same subject.

### 4.6. Normalization

The features extracted in the previous step do not share the same units, so it is very important to standardize them not only to remove their mean, but also scale them to a unit variance before feeding them into the machine learning model. Failing to do so can slow down training and even hinder the model learning process. This normalization process can be performed using the Equation (6):

(6) $$z=\frac{x-\mu }{\sigma }$$

where μ is the mean and σ is the standard deviation of the samples. That calculation has been easily measured with the help of the StandardScaler function available in the Python Scikit-learn library [44].

## 5. Experimental Setup

### 5.1. Dataset

**Figure 4:**
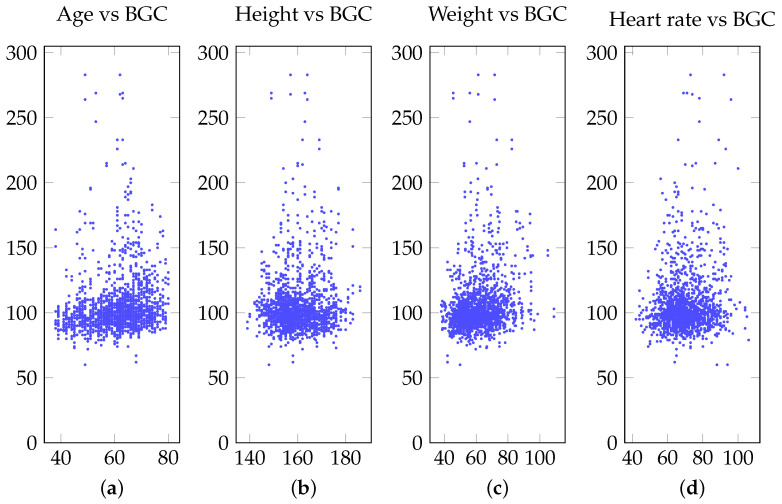
Distribution of the blood glucose concentration (BGC) with different profile information: (**a**) age; (**b**) height; (**c**) weight; (**d**) heart rate.

Several public ECG datasets are available for research purposes, especially at the Physionet bank [45]. Unfortunately, in order to detect hyperglycemia, the dataset must include not only ECG data, but also glucose concentration measurements taken at the same time as the ECG was being recorded. In addition, in order to have a robust model, a large amount of data should be available for model training and testing. In this study, we used a large, novel ECG dataset that contains not only ECG recordings and blood glucose concentration, but also profile information such as age, gender, height, weight, and heart rate. The distribution of blood glucose concentration across those different profiles can be seen in Figure 4.

The database was collected by the Research Center for Applied Sciences, Academia Sinica, Taiwan based on the following protocol:

- Each subject participated in two sequential recording sessions, both taken in the morning.
- Each session consisted of the recording of a 60-s single-lead ECG and blood glucose concentration.
- ECG was acquired using Analog AD-8232 with a sampling rate of 1000 Hz [46].
- Blood glucose concentration was measured using Accu-Chek Mobile blood glucose monitoring system [47].

**Figure 5:**
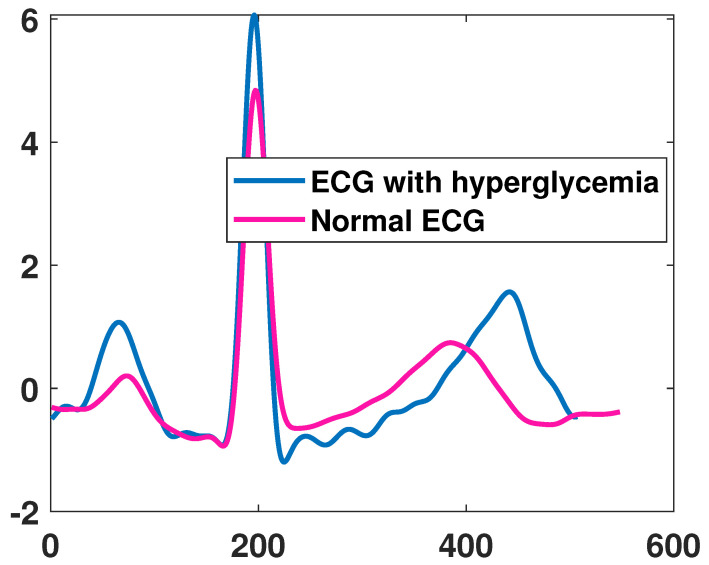
ECG signal from a person with hyperglycemia and without hyperglycemia.

A total of 1119 subjects participated, including 386 females and 733 males with age varying from 38 to 80 years old. They were not required to fast and their overall health status was not disclosed. For this work, each of the ECG recordings was analyzed and the ones with low quality were discarded, resulting in dataset of 1963 recordings. The ones from subjects with glucose concentration higher than 100 mg/dL were labeled with hyperglycemia. An example of ECG recordings from a subject with hyperglycemia and non-hyperglycemia can be seen in Figure 5. Both curves are similar in shapes and thus a simple visual inspection of such ECG would not be sufficient to identify if the patient is with hyperglycemia, which is possible but extracting the fiducial features as proposed in the work.

### 5.2. Hardware and Software

A computer with processor Intel(R) Core(TM) i9-9900K 3.60 GHz, 16 GB RAM, and 64-bit Windows OS was used for the simulations. All the work was performed using Kubios HRV Premium software [48], Python programming language, BioSPPy (Biosignal Processing in Python), NeuroKit framework for Python, Jupyter notebooks, Keras, and TensorFlow.

### 5.3. Training and Testing

The 1963 ECG readings were segmented in cardiac cycles and then an equal proportion of hyperglycemia state and non-hyperglycemia state were selected, resulting in a dataset of 68,274. As mentioned before, the outlier removal process purged 16,756 samples, leaving a net of 51,518 samples to be used in the model. An 80/20 split was used to create the training/testing dataset; therefore, the training dataset contained 41,214 samples and the 10,304 testing samples, with almost equal representation of hyperglycemia and non-hyperglycemia samples.

### 5.4. Models and Metrics

Several machine learning models and configurations have been explored, including logistic regression, support vector machine (linear, gaussian, polynomial) and deep neural network (DNN). Three performance metrics were used to evaluate the hyperglycemia detection performance: true positive rate (TPR), false positive rate (FPR), and the area under the curve (AUC). FPR or (1-Specificity) is the percentage of healthy ECG that is wrongly classified as hyperglycemia whereas TRP or sensitivity is the percentage of hyperglycemic events that was successfully classified as hyperglycemia. AUC of the receiver operating characteristic (ROC) curve was the performance metric used to compare the models since in binary classification problems, such as these, the threshold used to distinguish between the two output labels (hyperglycemia and non-hyperglycemia) have a direct impact in performance metrics. The ROC plots the model performance in terms of TPR versus FPR across different thresholds. The area of these curves thus provides a combined performance measurement of all these thresholds. We also used sensitivity and specificity as another metric for classification performance. Sensitivity measures how often a test correctly generates a positive result for people who have the hyperglycemia condition being tested for, whereas specificity measures a test’s ability to correctly generate a negative result for people who do not have hyperglycemia. Extensive simulations of different DNN architectures were also performed in order to identify optimal hyperparameters. For non-ANN models, the simulations were configured with a maximum number of iterations of 10,000. For ANN simulations, the models were trained with 1000 epochs and early stopping when no loss improvement has been achieved in the last 100 epochs. In order to make sure the DNN does not over-train, validation has been used as an early stopping method. The training optimizer used a stochastic gradient descent (SGD) with learning rate 0.0001, which was reduced by half every time no improvement was obtained after 20 sequential epochs.

## 6. Experimental Result

Table 3 summarizes the simulations with the best performance for the different models. A comprehensive list of all models tested and their respective performance can be seen in Appendix A.

**Figure 6:**
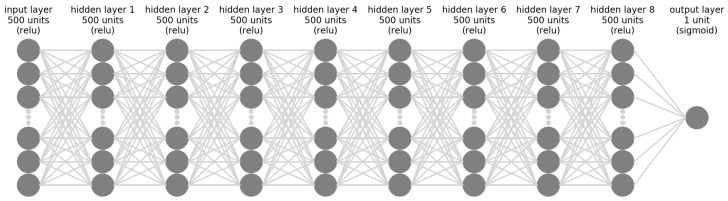
Deep neural network architecture that provided the best performance.

**Figure 7:**
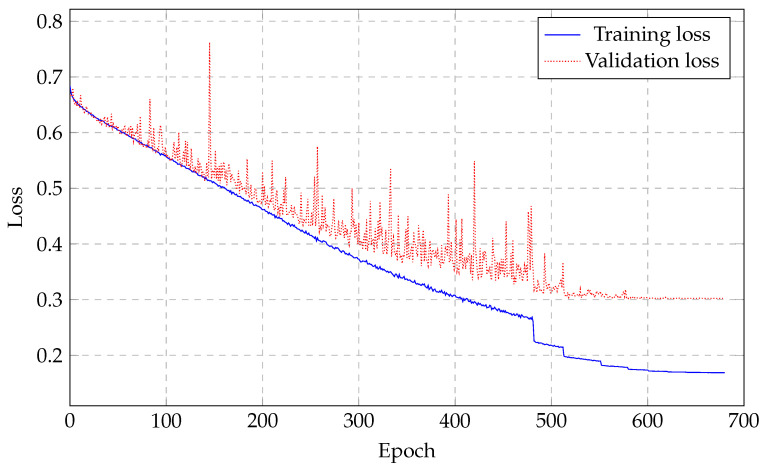
The 10-layer DNN training and validation loss. It shows that the network has stopped learning 500 epoch in which the validation is not degraded.

The deep neural network with 10 layers and 500 units per layer, except for the output layer, provided the best performance among all models simulated. The network architecture can be seen in Figure 6. The loss training is illustrated in Figure 7 and it is noticeable how the network stopped learning right after the 500 epoch mark with the validation loss not improving anymore. After training, the testing dataset is then used to validate the performance of our proposed DNN architecture. For these, the whole dataset, which included both hyperglycemia data and non-hyperglycemia data, was used.

**Figure 8:**
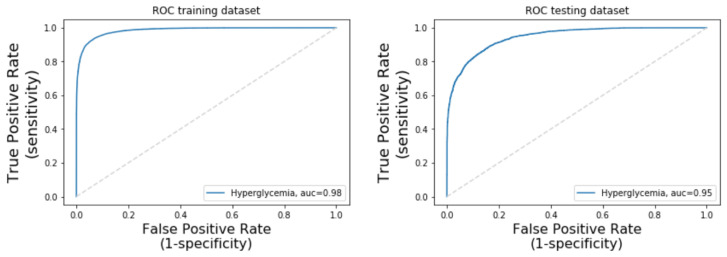
The 10-layer DNN ROC and AUC.

The model presented a training AUC of 98.44% and testing AUC of 94.53% as can be seen in the ROC curves in Figure 8.

A 10 k-fold cross validation was also performed in order to verify the performance consistency of the model, resulting in an average AUC of 93.65%. The AUC for each k-fold round can be seen in Table 4.

The proposed model was also compared against the best model identified in the literature. Since the work presented by [6] was developed based on different features and dataset, we replicated their 3-layer ANN model with a few adjustments and using our dataset. For each of the 1963 ECG readings we extracted the same 16 features described in Table 1. The time and frequency domain features were extracted using the Kubios HRV Premium software. We created the 3-layer ANN with nine nodes in the hidden layer. Instead of using the Levenberg–Marquard algorithm for training and hyperbolic tangent sigmoid (Tansig) as transfer function, we used stochastic gradient descent (SGD) for training and the hyperbolic tangent (tanh) as the transfer function since the originals were not available in Keras. The simulated model resulted in a testing sensitivity of 65.64%, specificity of 56.21%, and AUC of 61.68%. A comparison between our model and the proposed by [6], with the adjustments detailed above, can be seen in Table 5.

The 10-layer DNN proposed in this work represents not only an relative performance improvement of 53% but also a demonstration that an end-to-end DNN approach has the potential to be used on ECG-based hyperglycemia detection system.

### Results Discussion

Additionally, heart rate, age, weight, and height profiling studies may also show important characteristics to substantiate and understand the effect of hyperglycemia as previous studies have shown. As can be seen in Figure 4, there is no significant correlation between blood glucose concentration and profile information such as heart rate, age, weight and height. In other words, blood sugar can affect people of any age, weight, height, and heart rate. Our proposed non-invasive continuous glucose monitoring system will also help each user keep control over his/her blood sugar and prevent complications. Even though we found that heart rate variability has no direct impact on hyperglycemia, several research suggested using heart rate variability to detect diabetes. Swapna et al. [49] heart rate variability (HRV) derived from ECG signal as a source of diabetes detection. They employed long short-term memory (LSTM) and convolutional neural network (CNN) to extract HRV features; followed by SVM classification. A total of 20 subjects with 10 min recording from normal and diabetes groups are collected. They claimed 95.7% accuracy is obtained when they applied CNN 5-LSTM with the SVM network. Singh et al. [50] also used heart rate variability (HRV) to detect blood glucose levels. In the Framingham Offspring Study, One thousand nine hundred nineteen with a mix of men and women participated. HRV variables features that were included in their study were standard deviation normal RR intervals, high frequency (HF, 0.15 to 0.40 Hz) and low-frequency (LF, 0.04 to 0.15 Hz) power, and LF/HF ratio. Similar to [50], Faust et al. [51] used high frequency (HF), and low-frequency (LF) feature set from RR interval with 15 patients with diabetes; 15 healthy volunteers. An approximate entropy (ApEn) was employed to measure distinction between regularity and irregularity of HRV. In recent work, Li et al. [52] they used ECG signal with 21 subjects to monitor three glucose ranges using CNN where 87.94% accuracy in low glucose level, 69.36% accuracy in moderate glucose level, and 86.39% in high glucose level was reported. They did not identify hyperglycemia in their study. Moreover, most work on hyperglycemia detection used limited feature sets including *HR*. The drawback of work is that heart rate variability will change based on different conditions such as exercise, emotional, walking, etc. So it would be difficult to judge if the changes are due to hyperglycemia or other conditions.

## 7. Challenge

Collecting ECG signals is a challenging task as it is sensitive to various environmental factors that will impact on the quality of data. Thus, inaccurate ECG acquisition may lead to a wrong prediction and accordingly affect clinical decision. Moreover, in this work we used a fiducial feature extraction technique to distinguish between hyperglycemia and non-hyperglycemia; however, the fiducial feature extraction method is sensitive to noise and may lead to a wrong fiducial detection.

## 8. Conclusions

Automated continuous hyperglycemia detection is a real need for patients who need to frequently monitor their blood glucose levels such as type 1, 2 diabetic people or pregnant women with gestational diabetes. ECG-based hyperglycemia identification is an non-invasive method that provides continuous high blood glucose level monitoring. The proliferation of consumer wearable devices with ECG reading capabilities such as smart watches, wristbands, and even handheld ECG readers provide an environment where ECG acquisition is becoming accessible to everyone. The recent advancements in low-cost wearable sensor technologies enable the use of ECG-based continuous glucose monitoring that is quick, painless, and easy, without the need of extra hardware or devices being placed in the patient.

To this end, various machine learning models and feature extraction techniques have been adopted to determine optimal parameters so that better accuracy can be obtained. This work presented a novel fiducial feature extraction method with 10-layer deep neural network. Our proposed deep learning model achieved a 94.53% AUC, 87.57% sensitivity, and 85.04% specificity, representing an relative improvement of 53% versus the best model found in the literature.

For future work, the extension of this model to detect hypoglycemia and even the shift from a classification model to a glucose concentration prediction (regression) could be sought after.

## Figures and Tables

**Table 1:** Features list used by Nguyen et al. [6] to calculate hyperglycemia.

#	Feature	Type
1	*HR*	Intervals
2	*PR*	Intervals
3	*QTc*	Intervals
4	*RTc*	Intervals
5	*TpTec*	Intervals
6	Mean RR interval	Time-domain
7	Standard deviation of the RR Interval index (SDNN)	Time-domain
8	Root mean square of successive RR interval differences (RMSSD)	Time-domain
9	Percentage of consecutive RR intervals that differ by more than 50 ms (pNN50)	Time-domain
10	HRV triangular index (HRVi)	Time-domain
11	Baseline width of the RR interval histogram evaluated through triangular interpolation (TINN)	Time-domain
12	Very low frequency (VLF)	Frequency-domain
13	Low frequency (LF)	Frequency-domain
14	High frequency (HF)	Frequency-domain
15	Total spectral power (TotalPw)	Frequency-domain
16	LF/HF ratio	Frequency-domain

**Table 2:** Features extracted from ECG.

#	Feature	#	Feature
1	*PQ* length	10	*QR* slope
2	*PQ* slope	11	*QS* length
3	*PR* length	12	*QS* slope
4	*PR* slope	13	*QT* length
5	*PS* length	14	*QT* slope
6	*PS* slope	15	*RS* length
7	*PT* length	16	*RS* slope
8	*PT* slope	17	*RT* length
9	*QR* length	18	*RT* slope

**Table 3:** Models performance.

Model	AUC
10-layer DNN	94.53%
Logistic Regression (C = 5)	62.44%
SVM Linear (C = 50)	58.99%
SVM Polynomial (d = 6)	56.36%
SVM Gaussian (C = 2)	52.03%

**Table 4:** The 10 k-fold cross validation (10-layer DNN).

k	AUC	k	AUC
1	96.98%	6	97.17%
2	97.23%	7	97.43%
3	96.40%	8	98.23%
4	97.34%	9	96.94%
5	96.03%	10	95.49%

**Table 5:** Model comparison.

	Sensitivity	Specificity	AUC
10-layer DNN	87.57%	85.04%	94.53%
3-layer ANN [6] modified	65.64%	56.21%	61.68%

## Data Availability

Restrictions apply to the availability of these data. Data was obtained from Research Center for Applied Sciences, Academia Sinica, Taiwan and are available from the authors with the permission of Research Center for Applied Sciences, Academia Sinica, Taiwan.

## Appendix A. Detailed Models Simulations Results

The Table A1 below shows the performance, measured as the Area Under the Curve (AUC) of the testing dataset for different non-ANN models and C (or degree for SVN Polynomial) parameters.

**Table A1:** AUC values for different models and C/degree parameter.

C or Degree	Logistic Regression	SVM Linear	SVM Gaussian	SVM Polynomial
0.001	61.45%	58.02%	18.60%	-
0.01	62.09%	53.86%	18.16%	-
0.1	61.87%	42.17%	17.61%	-
1	62.37%	57.36%	47.90%	55.92%
2	61.87%	44.01%	52.03%	55.14%
3	61.87%	57.57%	52.03%	42.74%
4	61.92%	43.47%	52.03%	48.17%
5	62.44%	42.22%	52.03%	41.85%
6	61.95%	41.88%	52.03%	56.36%
7	61.91%	41.96%	52.03%	50.15%
8	62.43%	44.88%	52.03%	50.00%
9	61.85%	50.16%	52.03%	50.00%
10	62.39%	41.37%	52.03%	50.00%
20	61.86%	57.93%	52.03%	50.00%
30	61.84%	48.64%	52.03%	50.00%
40	61.90%	41.66%	52.03%	50.00%
50	62.44%	58.99%	52.03%	50.00%
60	61.86%	57.56%	52.03%	50.00%
70	61.87%	56.22%	52.03%	50.00%
80	61.93%	51.63%	52.03%	50.00%
90	61.92%	56.93%	52.03%	50.00%
100	62.37%	42.46%	52.03%	50.00%

For ANN models, Table A2 below shows the AUC for different number of layers and number of units per layer.

**Table A2:** AUC values for different number of layers and units per layer.

	# of Units per Layer (exc. Output Layer)	100	200	300	400	500
# of Layers						
2		49.96%	50.00%	50.00%	79.64%	50.00%
3		79.34%	50.00%	50.00%	50.00%	50.00%
4		80.46%	49.99%	88.68%	50.00%	89.25%
5		83.64%	87.02%	88.57%	90.76%	50.00%
6		82.94%	89.85%	91.68%	91.06%	92.53%
7		85.69%	90.78%	91.94%	92.43%	93.20%
8		86.57%	89.49%	92.76%	92.26%	93.44%
9		88.81%	89.96%	92.80%	92.91%	93.59%
10		89.06%	91.88%	92.29%	94.34%	94.53%
